# Factors Associated With Using the COVID-19 Mobile Contact-Tracing App Among Individuals Diagnosed With SARS-CoV-2 in Amsterdam, the Netherlands: Observational Study

**DOI:** 10.2196/31099

**Published:** 2022-08-24

**Authors:** Feiko Ritsema, Jizzo R Bosdriesz, Tjalling Leenstra, Mariska W F Petrignani, Liza Coyer, Anja J M Schreijer, Yvonne T H P van Duijnhoven, Janneke H H M van de Wijgert, Maarten F Schim van der Loeff, Amy Matser

**Affiliations:** 1 Department of Infectious Diseases GGD Amsterdam Amsterdam Netherlands; 2 Department of Internal Medicine Amsterdam Institute for Infection and Immunity Amsterdam UMC, Academic Medical Center Amsterdam Netherlands; 3 Julius Center for Health Sciences and Primary Care University Medical Center Utrecht Utrecht University Utrecht Netherlands

**Keywords:** COVID-19, contact tracing, mobile contact tracing app, pandemic, mHealth, digital health, contact tracing app, mobile applications, health applications, public health, surveillance

## Abstract

**Background:**

Worldwide, efforts are being made to stop the COVID-19 pandemic caused by SARS-CoV-2. Contact tracing and quarantining are key in limiting SARS-CoV-2 transmission. Mathematical models have shown that the time between infection, isolation of cases, and quarantining of contacts are the most important components that determine whether the pandemic can be controlled. Mobile contact-tracing apps could accelerate the tracing and quarantining of contacts, including anonymous contacts. However, real-world observational data on the uptake and determinants of contact-tracing apps are limited.

**Objective:**

The aim of this paper is to assess the use of a national Dutch contact-tracing app among notified cases diagnosed with SARS-CoV-2 infection and investigate which characteristics are associated with the use of the app.

**Methods:**

Due to privacy regulations, data from the app could not be used. Instead, we used anonymized SARS-CoV-2 routine contact-tracing data collected between October 28, 2020, and February 26, 2021, in the region of Amsterdam, the Netherlands. Complete case logistic regression analysis was performed to identify which factors (age, gender, country of birth, municipality, number of close contacts, and employment in either health care or education) were associated with using the app. Age and number of close contacts were modelled as B-splines due to their nonlinear relationship.

**Results:**

Of 29,766 SARS-CoV-2 positive cases, 4824 (16.2%) reported app use. Median age of cases was 41 (IQR 29-55) years, and 46.7% (n=13,898) were male. In multivariable analysis, males (adjusted odds ratio [AOR] 1.11, 95% CI 1.04-1.18) and residents of municipalities surrounding Amsterdam were more likely to use the app (Aalsmeer AOR 1.34, 95% CI 1.13-1.58; Ouder-Amstel AOR 1.96, 95% CI 1.54-2.50), while people born outside the Netherlands, particularly those born in non-Western countries (AOR 0.33, 95% CI 0.30-0.36), were less likely to use the app. Odds of app use increased with age until the age of 58 years and decreased sharply thereafter (*P*<.001). Odds of app use increased with number of contacts, peaked at 8 contacts, and then decreased (*P*<.001). Individuals working in day care, home care, and elderly nursing homes were less likely to use the app.

**Conclusions:**

Contact-tracing app use among people with confirmed SARS-CoV-2 infection was low in the region of Amsterdam. This diminishes the potential impact of the app by hampering the ability to warn contacts. Use was particularly low among older people, people born outside the Netherlands, and people with many contacts. Use of the app was also relatively low compared to those from some other European countries, some of which had additional features beyond contact tracing, making them potentially more appealing. For the Dutch contact-tracing app to have an impact, uptake needs to be higher; therefore, investing more into promotional efforts and additional features could be considered.

## Introduction

The COVID-19 pandemic, caused by SARS-CoV-2, has had a major impact. Two years into the pandemic, as of December 2021, over 260 million people have been infected worldwide, of whom more than 5 million have died [1]. Large-scale control measures are necessary to limit transmission of an emerging infectious disease such as COVID-19, for which a vaccine or treatment is (initially) unavailable [2]. Nonpharmaceutical interventions have been implemented by many countries, including face masking, physical distancing, travel restrictions, large-scale testing, and contact tracing [3]. To prevent the onward transmission of SARS-CoV-2, it is key to identify, test, and isolate infectious cases.

Contact tracing is a targeted approach to identify individuals who have been in close contact with confirmed cases [2]. The contacts of cases should be quarantined as soon as possible because the incubation period is short, and individuals can become infectious even before the onset of symptoms [4]. Contact tracing is a labor-intensive and time-consuming process. Its effect largely depends on the speed of contact tracing and the proportion of contacts that index cases are willing and able to identify from the start of probable infectiousness [5]. This is complicated by the fact that many of these contacts might be anonymous. Mathematical models have shown that the time between infection and isolation of cases, on the one hand, and quarantining of contacts, on the other, are the most important components that determine whether the pandemic can be controlled [6-8]. They also show that reducing delays in testing and contact tracing could reduce the spread of the virus, especially when there is no delay between case notification and quarantining of contacts. The models suggest that tracing apps for mobile phones have the potential to speed up the contact-tracing process and help identify unknown contacts, thereby significantly curbing SARS-CoV-2 spread [7-9]. However, these mathematical models rely on several assumptions, some of which might be violated by real world data, making it necessary to complement these studies with observational research.

Many countries have implemented tracing apps to identify and notify contacts of SARS-CoV-2 cases with various levels of success [10-17]. This fits in with a more generalized trend of increasing use of mobile apps for tracking and managing many aspects of health and behavior, providing users with more (sense of) control [18]. In the Netherlands, a tracing app developed by the Dutch government (CoronaMelder) was launched on October 10, 2020. The Dutch app uses Bluetooth to register other mobile phones on which the app is installed, their Bluetooth is active, and are within a 1.5-meter radius for at least 15 minutes. Data are stored locally on mobile phones for 14 days. When someone tests positive for SARS-CoV-2, the Public Health Service (PHS) will initiate contact tracing. As part of that process, the index case is asked whether they are using the app and are willing to notify the contacts that were registered by the app via the app. The registered contacts will subsequently receive a notification that they have been close to someone with a SARS-CoV-2 infection and the date on which this happened. In this notification, the app users are advised to quarantine themselves with immediate effect and to get tested. From October 10, 2020, to December 1, 2020, app users who received a notification were only allowed to be tested free of charge at a PHS facility if they were symptomatic. However, from December 1, 2020, onwards, asymptomatic users were also allowed free testing from the 5th day after the most recent exposure listed in the app notification.

Introduction of the app required an amendment to Dutch law [19] and generated much political and societal discussion about safeguarding the privacy of users. Controlling the spread of SARS-CoV-2 and protecting personal health are mentioned as main determinants of the willingness to use contact-tracing apps [20,21]. Conversely, safety and privacy concerns were associated with lower willingness to use the app. In general, 41% to 66% of participants were willing to use the app [20,21], which could be sufficient to reduce SARS-CoV-2 spread [8]. These figures are comparable to other Western European countries, where over 40% of participants said they would definitely install such an app, and an additional 35% of participants would probably install it [22]. However, the willingness to use an app might not lead to actual use. Nevertheless, reported app uptake numbers are encouraging (around 60% in Australia, Denmark, France, and the UK; 75% in the United States; and 90% in Japan [23]). More data are needed on actual app use in practice to complement theoretical models of app impact and willingness to use. Moreover, as research on other mobile health app has shown, there might be significant differences in uptake by age, income, education, health literacy, self-reported health, and intention to engage in healthy behavior [18]. To what extent those findings apply to an app such as CoronaMelder remains to be seen, since after installing it, no further active use of the app is required.

In this study, we therefore aimed to study the self-reported use of the Dutch CoronaMelder app and determinants of use in a real-life setting. As data from the app itself are not available due to privacy policies, we used data registered in the source and contact-tracing system after notification of a positive SARS-CoV-2 case instead. We evaluated which proportion of individuals who tested positive for SARS-CoV-2 (between October 28, 2020, and February 26, 2021, in the Amsterdam region) had used the mobile Dutch national contact-tracing app. Furthermore, we examined whether there were any significant differences in app uptake by several sociodemographic factors.

## Methods

### Population

In the Netherlands, SARS-CoV-2 tests are performed at publicly funded testing facilities of the PHS and hospitals, free of charge, or by commercial providers for a fee. SARS-CoV-2 is a notifiable infection, which means that all confirmed SARS-CoV-2 cases must be reported to the PHS regardless of where the testing took place. In this analysis, we included all adults (≥18 years old) who live in the Amsterdam region and were approached by the PHS of Amsterdam between October 28, 2020, and February 26, 2021, for contact tracing after a SARS-CoV-2 diagnosis.

Using data directly from the CoronaMelder app itself was not possible due to the anonymous nature of those data and privacy regulations. Therefore, we used data collected by PHS staff during routine contact tracing by phone and stored in HPZone (inFact UK Ltd). Routine procedure stipulates that PHS staff call persons diagnosed with SARS-CoV-2 (ie, cases) in the Netherlands to inform them about the diagnosis and isolation measures, and to initiate contact tracing. The case and a PHS staff member together systematically make an inventory of all identifiable persons that the case had been in contact with, 2 days prior to the date of symptom onset (if symptomatic) or positive test result (if asymptomatic). Moreover, PHS staff members are instructed to ask if the case used the CoronaMelder app, to note the answer in a standard format in a text field template in HPZone, and to activate the contact notification function of the app.

### Variables

Data for this study were extracted from HPZone and anonymized before analysis. We extracted age in years at symptom onset (for symptomatic cases) or at the time of initiating contact tracing (for asymptomatic individuals) as a continuous variable. Other variables of interest were gender, categorized as male and female (other or nonbinary was not available in the system, was regarded as missing, and was therefore excluded from the analyses), and the municipality of residence (Aalsmeer, Amstelveen, Ouder-Amstel, Diemen, Uithoorn, or Amsterdam). During contact tracing, contacts were categorized into household contacts, close contacts, or other contacts. For this study, we extracted the number of close contacts, defined as contacts with whom a case had been within 1.5 meters for more than 15 minutes, excluding household contacts. Self-reported country of birth was recorded and later categorized as the Netherlands, other Western country, or non-Western country, in accordance with the definition used by Statistics Netherlands [24]. Employment in health care was categorized as “not,” “hospital,” “nursing home for elderly,” “other 24-hour care home,” “in-home care,” and “other health care.” Employment in education was categorized as “not,” “day care,” “elementary school,” and “secondary or higher education.” Data on CoronaMelder app use was extracted using a regular expression (“Gebruik coronamelder:”) from the free text notes. For those who used the app, we also extracted data on the reason of requesting a SARS-CoV-2 test.

### Ethical Considerations

The medical ethics committee of the Amsterdam University Medical Centers deemed it not necessary to fully review the study, because the study does not fall under the scope of the Medical Research Involving Human Subjects Act (W20_432#20.479). No data from the app are used in this paper; therefore, the privacy regulations of the app were not reviewed for the purposes of this study, though they can be found on the web [25].

### Analysis

Differences in characteristics between individuals who reported to use the mobile app and individuals who did not were assessed with chi-squared tests for categorical variables and Kruskal-Wallis tests for continuous variables. Trends over time in data availability on app use, as well as app use itself, were tested with the Pettitt test. Logistic regression analyses were performed to identify determinants of mobile app use. First, in univariable models, we tested for each independent variable (age, gender, country of birth, municipality, number of close contacts, employment in health care, and employment in education) whether they were associated with the dependent variable—self-reported use of the CoronaMelder app. Second, we combined all aforementioned independent variables and the dependent variable in 1 multivariable model. Age in years and the number of close contacts were added as continuous variables. As these variables were found to have a nonlinear relation to the outcome variable in exploratory analysis and regressions, B-splines were used with respectively 4 and 2 knots and a degree of 2. Gender and self-reported use of the CoronaMelder app were added as dichotomous variables, and all other variables were added as categorical variables. A complete cases analysis was performed; cases with missing data were excluded from the analysis. Outliers in the continuous variables age in years (above 100 years old) and number of close contacts (more than 12 close contacts, 99th percentile) were removed. In sensitivity analysis, multiple imputation using Multivariate Imputation by Chained Equations was carried out to impute missing outcomes and independent variables [26,27]. Analysis was performed using the statsmodels library in Python3 (Python Software Foundation) [28].

## Results

**Figure 1:**
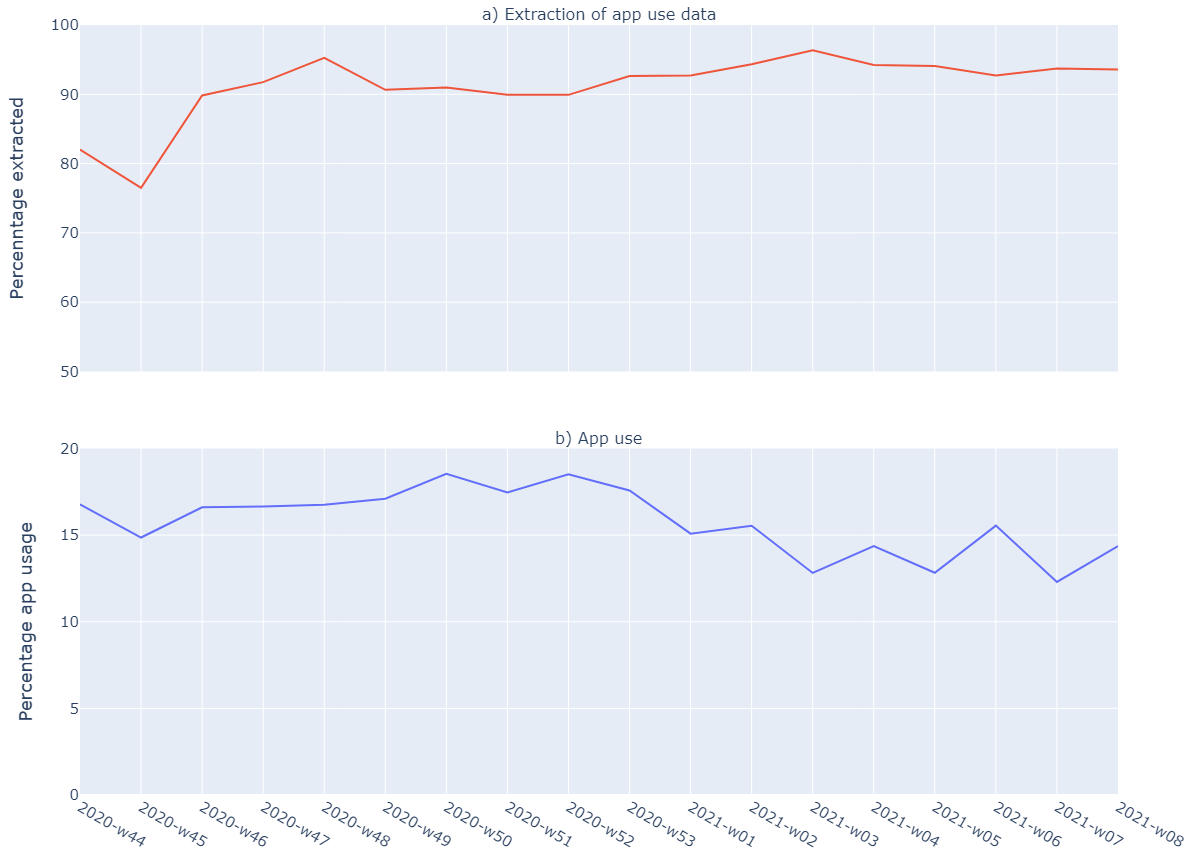
The percentage of cases with available data on the use of the contact tracing app (a) and the percentage of cases who used the mobile contact tracing app (b) by week (w) among SARS-CoV-2 positive cases in the region of Amsterdam (October 28, 2020, to February 26, 2021).

From October 28, 2020, until February 26, 2021, the PHS of Amsterdam contacted 34,591 cases who were ≥18 years old and lived in the region of Amsterdam for contact tracing. We excluded 3354 (9.7%) cases because data on app use were not available, 1310 (3.8%) cases because they had missing values in one of the explanatory variables (such as gender), and 161 (0.47%) cases because they were outliers (>100 years old or >12 close contacts). Missing data on app use were caused by either invalid entries (anything except “yes/no” and variants of this) or missing entries, and they were higher in the first weeks after the introduction of the app (Figure 1). Cases with missing data on app use were older and more often born in a non-Western country.

The median age of the 29,766 included cases was 41 years (IQR 29-55); 13,898 (46.7%) were male, and 18,798 (63.2%) were born in the Netherlands (Table 1). At the time of diagnosis, 4824 (16.2%) cases reported using the app. The number of cases reporting app use decreased significantly over time, especially after the first week of 2021—until January 4, 2021, a total of 5120 (17.2%) cases used the app, while this was 12,799 (14.3%) after that date (*P*=.001).

In total, 2494 (51.7%) out of 4824 app users and 15,442 (61.9%) out of 24,942 nonusers did not report any close contacts during the probable infectious period. The median number of reported close contacts among app users with at least one contact was 2 (IQR 1-4), and 2 (IQR 1-3) among nonusers. Among app users, 314 (6.5%) cases reported to have received a notification by the app that they had been in contact with a person diagnosed with SARS-CoV-2. The number of reported close contacts did not differ significantly between app users who received a notification and app users who did not receive a notification (*P*=.07, median 0; IQR 0-1; 90th percentile=3 for both groups). In total, 506/3227 (15.7%) individuals working in health care and 187/1154 (16.2%) individuals working in education used the app.

**Figure 2:**
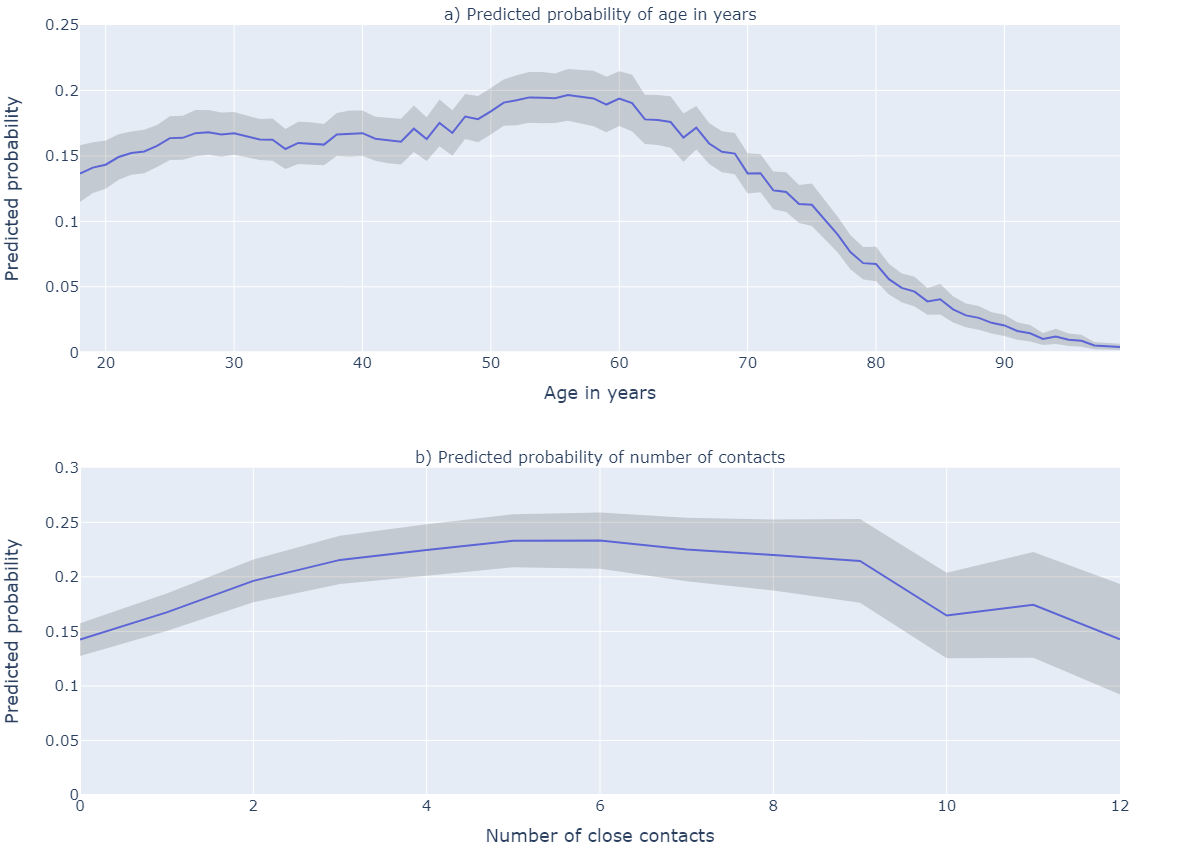
Predicted probability of reporting CoronaMelder app use by (a) age in years and (b) the reported number of close contacts, resulting from multivariable logistic regression analysis using B-splines among 29,766 SARS-CoV-2 positive cases in the region of Amsterdam (October 28, 2020, to February 26, 2021).

In multivariable logistic regression, the odds of reporting app use increased with increasing age (Figure 2a), until about the age of 58 years, after which the odds decreased sharply (*P*<.001). Men were slightly more likely to report app use than women (adjusted odds ratio [AOR] 1.11; 95% CI 1.04-1.18; Table 2). Cases who were born in other Western countries (AOR 0.74; 95% CI 0.65-0.84), and cases born in non-Western countries (AOR 0.33; 95% CI 0.30-0.36) were less likely to report app use compared with cases born in the Netherlands. Compared to cases living in the municipality of Amsterdam, cases living in most of the surrounding municipalities were more likely to report app use (eg, AOR 1.96; 95% CI 1.54-2.50 for cases living in Ouder-Amstel). Furthermore, there was a positive association between reporting more close contacts and reporting app use (Figure 2b), up to 8 reported close contacts, above which app use was less likely. Compared to cases not working in health care, cases working in elderly nursing homes (AOR 0.48; 95% CI 0.36-0.63) and home care (AOR 0.61; 95% CI 0.42-0.90) were less likely to report app use. The AOR for cases working in day care was 0.39 (95% CI 0.26-0.59) compared to cases not working in education.

The results after multiple imputation were similar to the results of complete case analyses (data not shown).

**Table 1:** Characteristics of individuals (≥18 years old) diagnosed with SARS-CoV-2 in the region of Amsterdam by reported mobile app use (October 28, 2020, to February 26, 2021).

Characteristics		Total^a^ (N=29,766)	App users (n=4824)	Nonusers (n=24,942)	*P* value^b^
Age (years), mean (IQR)		41 (29-55)	42 (29-54)	41 (29-55)	.89
**Gender, n (%)**					<.001
	Female	15,868 (53.3)	2437 (15.4)	13,431 (84.6)	
	Male	13,898 (46.7)	2387 (17.2)	11,511 (82.8)	
**Country of birth^c^, n (%)**					<.001
	Netherlands	18,798 (63.2)	3803 (20.2)	14,995 (79.8)	
	Non-Western	9116 (30.6)	730 (8.0)	8386 (92.0)	
	Other Western	1852 (6.2)	291 (15.7)	1561 (84.3)	
**Municipality, n (%)**					<.001
	Amsterdam	24,970 (83.9)	3832 (15.4)	21,138 (84.7)	
	Aalsmeer	852 (2.9)	197 (23.1)	655 (76.9)	
	Amstelveen	1921 (6.5)	408 (21.2)	1513 (78.8)	
	Diemen	895 (3.0)	145 (16.2)	750 (83.8)	
	Ouder-Amstel	340 (1.1)	99 (29.1)	241 (70.9)	
	Uithoorn	788 (2.7)	143 (18.2)	645 (81.9)	
Median close contacts, mean (IQR)		0 (0-1)	0 (0-2)	0 (0-1)	<.001
**Close contacts, n (%)**					<.001
	0	17,936 (60.3)	2494 (13.9)	15,442 (86.1)	
	1-3	9133 (30.7)	1736 (19.0)	7397 (81.0)	
	4-6	2024 (6.8)	449 (22.2)	1575 (77.8)	
	>6	673 (2.3)	145 (21.6)	528 (78.5)	
**Employment in health care, n (%)**					<.001
	No	26,539 (89.2)	4318 (16.3)	22,221 (83.7)	
	Hospital	845 (2.8)	162 (19.2)	683 (80.8)	
	Nursing home for elderly	664 (2.2)	57 (8.6)	607 (91.4)	
	Other 24-hour care home	331 (1.1)	49 (14.8)	282 (85.2)	
	Home care	261 (0.9)	31 (11.9)	230 (88.1)	
	Other health care	1126 (3.8)	207 (18.4)	919 (81.6)	
**Employment in education, n (%)**					<.001
	No	28,612 (96.1)	4637 (16.2)	23,975 (83.8)	
	Yes, day care	340 (1.1)	26 (7.7)	314 (92.4)	
	Yes, elementary school	637 (2.1)	130 (20.4)	507 (79.6)	
	Yes, secondary or higher education	177 (0.6)	31 (17.5)	146 (82.5)	

^a^From the total sample, the following have been excluded: 3354 cases because of missing data on app use, 1310 cases because of missing values on an independent variable, and 161 cases because of outliers on continuous variables.

^b^*P* values for differences between app users and nonusers were assessed with Kruskal-Wallis tests for age and number of close contacts, and with chi-squared tests for all other variables.

^c^For the categorization of country of birth into non-Western or other Western, the definition from Statistics Netherlands was used [24].

**Table 2:** Factors associated with mobile app use among 29,283 individuals (≥18 years old) diagnosed with SARS-CoV-2 in the region of Amsterdam (October 28, 2020, to February 26, 2021).

Characteristics		OR^a^ (95% CI)^b^	*P* value	AOR^c^ (95% CI)^b^	*P* value
Age^d^		—^e^	<.001	—	<.001
**Gender**			<.001		.002
	Female	1		—	
	Male	*1.14 (1.07-1.22)*		*1.11 (1.04-1.18)*	
**Country of birth^d^**			<.001		<.001
	Netherlands	1		—	
	Other Western	*0.74 (0.65-0.84)*		*0.74 (0.65-0.84)*	
	Non-Western	*0.34 (0.32-0.37)*		*0.33 (0.30-0.36)*	
**Municipality**			<.001		<.001
	Amsterdam	1		1	
	Aalsmeer	*1.66 (1.41-1.95)*		*1.34 (1.13-1.58)*	
	Amstelveen	*1.49 (1.33-1.67)*		*1.43 (1.27-1.61)*	
	Diemen	1.07 (0.89-1.28)		1.02 (0.85-1.23)	
	Ouder-Amstel	*2.27 (1.79-2.87)*		*1.96 (1.54-2.50)*	
	Uithoorn	*1.22 (1.02-1.47)*		1.03 (0.85-1.25)	
Number of close contacts^f^		—	<.001	—	<.001
**Employment in health care**			<.001		<.001
	No	1		1	
	Hospital	*1.22 (1.03-1.45)*		1.02 (0.85-1.22)	
	Nursing home for elderly	*0.48 (0.37-0.64)*		*0.48 (0.36-0.63)*	
	Other 24-hour care home	0.89 (0.66-1.21)		0.78 (0.57-1.06)	
	Home care	0.69 (0.48-1.01)		*0.61 (0.42-0.90)*	
	Other health care	1.16 (0.99-1.35)		0.95 (0.81-1.12)	
**Employment in education**			<.001		<.001
	No	1		1	
	Yes, day care	*0.43 (0.29-0.64)*		*0.39 (0.26-0.59)*	
	Yes, elementary school	*1.33 (1.09-1.61)*		1.07 (0.88-1.31)	
	Yes, secondary or higher education	1.1 (0.74-1.62)		0.91 (0.61-1.35)	

^a^OR: odds ratio.

^b^Significant associations are italicized.

^c^AOR: adjusted odds ratio.

^d^For the categorization of country of birth into non-Western or Other Western, the definition from Statistics Netherlands was used [24].

^e^Not applicable.

^f^Variables modelled as B-splines (Figure 2).

## Discussion

### Principal Findings

In this study, we found that fewer than 1 in 6 individuals diagnosed with SARS-CoV-2 in the region of Amsterdam reported using the CoronaMelder contact-tracing app. As 24,942 (84%) out of 29,766 cases were not using the app, their close contacts could never receive a notification through the app, even though they might have installed it themselves. Only 6.5% (1935/29,766) of the positive cases with the app had received an app notification themselves. Reporting app use was associated with being middle-aged, having a few (ie, 3-8) close contacts during the infectious period, living in municipalities surrounding Amsterdam (rather than the city itself), and being born in the Netherlands. App use was less often reported by individuals with more than 8 close contacts and individuals who are born outside the Netherlands.

### Limitations

Caution is warranted when interpreting these results and the potential explanations and implications. Moreover, these results cannot be directly extrapolated to the general population, including those who did not test positive for SARS-CoV-2. According to national data, over 4.5 million people have downloaded the app during the study period [29], which is approximately 26% of the Dutch population. In our study population of cases, however, this percentage was only 16% (4824/29,766). It is possible that the cases included in our sample represent a population that is less likely to take any preventive measures. It is also possible that PHS staff did not consistently ask cases about app use, as Amsterdam has been a region with high infection numbers, leading to high work pressure for the contact-tracing team. On the other hand, the national number is a cumulative number that does not account for app removals or inactivation, multiple app downloads by one person, or underreporting of app use during contact tracing, while the number in our study represents prevalent use. A second limitation is that the routine PHS data were not collected for the purpose of scientific research. This limits the number of variables and thus the potential to explain our observations. Furthermore, ascertainment bias may have been introduced because data may not have been collected consistently and uniformly. However, sensitivity analysis showed that bias caused by missing data was very small.

### Comparison With Prior Work

In Dutch acceptability studies performed prior to the introduction of the app, in April 2020, younger individuals reported to be more willing to download the app once available [20,21]. A survey performed in France, Germany, Italy, the United Kingdom, and the United States showed the same age trend [22]. Willingness to download the app was associated with positive attitudes toward technology and with fear for COVID-19 [21]. However, in our study, middle-aged individuals were more likely to use the app compared to younger individuals. Fear for COVID-19 might have played a role in these older age groups, in line with their higher risk of more serious disease once infected. Conversely, the absence of fear, privacy concerns, and a lower willingness to obey COVID-19 control measures might have been more important among younger individuals. The oldest individuals in our study were less likely to use the app, which may relate to lower smartphone and app usage among elderly people in general [18]. This is supported by another evaluation of the same CoronaMelder app, which showed that elderly people had problems with understanding why, when, and how to use the app [30].

Cases living in the municipalities of Amsterdam or Diemen, the latter being geographically strongly connected to Amsterdam, were less likely to use the app compared with cases in the surrounding municipalities. Improving app use in more densely populated urban settings might be worthwhile because the app is especially useful to identify anonymous close contacts who cannot be traced otherwise. Additionally, we found strong associations with being born outside the Netherlands and not using the app. If national app usage trends reflect those found in our sample, this would be worrisome given that previous studies in Amsterdam and internationally have shown that some ethnic minority groups are disproportionately affected by SARS-CoV-2 [31-36]. Cultural differences or distrust in the authorities may underlie this observation, but other more practical issues might be important as well. Even though the app itself and information on the CoronaMelder app website are available in 10 different languages, language barriers might still exist, and communication about the app might not reach all groups. Unfortunately, the routine data used in this study do not contain information on language skills or parental birth country, and thus we cannot investigate app use among second-generation immigrants. Further research in this group is therefore needed to reduce health inequalities between ethnic groups [35,36].

While the likelihood of using the app increased with the number of close contacts in the range of 0 to 8 close contacts, it decreased with higher number of contacts. The advantages of using an app for contact tracing include speed, the fact that anonymous close contacts can be reached, and that there is no recall bias, which is especially beneficial among individuals with many contacts. Thus, it might be worthwhile to study barriers for use and promote app use among individuals with many close contacts.

Lastly, we saw moderate differences in app use among people working in high-risk professions during which many contacts may be unavoidable. In the health care sector, precautions are taken to prevent infection (eg, use of personal protective equipment). Using the app during working hours may result in *false* notifications that are indistinguishable from notifications after real risk contacts. This might explain why people working in nursing homes and home care were using the app less often compared to individuals not working in health care. However, a pause button was introduced to the app (to be used in situations such as when the phone is left in a locker) to allow people to keep the app but reduce the chance of receiving *false* notifications [37]. Place of work as a reason for not downloading the app was mentioned in a survey in the United Kingdom [38]. Individuals working in day care centers were also less likely to have the app. For this group, the app could be of added value because they encounter parents of children without full protection.

If contact-tracing apps are used efficiently and uptake is high, they have the potential to speed up contact tracing, identify contacts that would otherwise go unnoticed, and prevent infections. For instance, the app of the National Health Service in the United Kingdom has been downloaded by 49% of the eligible population with compatible smartphones [39], which is >30% of the total population. A modelling study showed that this app averted about one case per index case willing to send the notification to their contacts [39]. The percentage of the population who downloaded tracing apps was also high in countries such as Germany, Switzerland, and Finland (ie, 32%-45%), but much lower in Spain, Italy, and France (ie, 15%-19%) [40]. This high level of adoption in some countries could be driven by the fact that the National Health Service app and the German app are among several apps that combine the tracing function with other features such as local area risk indicators and a link for booking a test, or by differences in promotion efforts. The Dutch Ministry of Health decided not to equip the CoronaMelder app with such additional features that might appeal to users, and soon after introduction, it stopped actively promoting app use. Instead, the Dutch Ministry of Health developed a second national app to function as a COVID-19 passport, registering vaccinations, recovery from infection, and negative test results. Combining the contact-tracing app and the corona passport app might have increased use of the tracing app. This knowledge, combined with the observed low uptake of the app in our sample, suggests that, to yield its potential effect on the control of the COVID-19 epidemic, the app needs to be used by more people. Based on our findings, app promotion efforts should particularly target younger individuals, individuals with >8 close contacts, and individuals who are not born in the Netherlands.

### Conclusions

This study shows that app use is low; only 4824 (16.2%) out of 29,766 individuals who tested positive for SARS-CoV-2 in the Amsterdam region. Moreover, we observed significant differences in app uptake by sociodemographic factors. Elderly persons, women, people not born in the Netherlands, and those either reporting none or many close contacts were less likely to have installed the CoronaMelder app. If confirmed in a nationally representative sample, this would mean the app is unlikely to have the impact on SARS-CoV-2 spread it could potentially have. Moreover, app uptake seems to be lower in certain subgroups of the population, indicating that more targeted efforts to improve uptake are necessary.

## Abbreviations

AOR: adjusted odds ratio
PHS: Public Health Service
